# Cell Death Pathways: a Novel Therapeutic Approach for Neuroscientists

**DOI:** 10.1007/s12035-017-0793-y

**Published:** 2017-10-19

**Authors:** G. Morris, A. J. Walker, M. Berk, M. Maes, B. K. Puri

**Affiliations:** 1 Bryn Road Seaside 87, Llanelli, Wales, SA15 2LW UK; 2 School of Medicine, Deakin University, Geelong, 3220 Australia; 3 The Centre for Molecular and Medical Research, School of Medicine, Deakin University, P.O. Box 291, Geelong, 3220 Australia; 4 Department of Clinical Medicine and Translational Psychiatry Research Group, Faculty of Medicine, Federal University of Ceará, Fortaleza, CE 60430-040 Brazil; 5 IMPACT Strategic Research Centre, School of Medicine, Deakin University, P.O. Box 291, Geelong, 3220 Australia; 6 Orygen Youth Health Research Centre and the Centre of Youth Mental Health, The Florey Institute for Neuroscience and Mental Health and the Department of Psychiatry, University of Melbourne, Parkville, 3052 Australia; 7 Department of Psychiatry, Chulalongkorn University, Bangkok, Thailand; 8 Department of Medicine, Hammersmith Hospital, Imperial College London, London, W12 0HS UK

**Keywords:** Apoptosis, Necroptosis, Ferroptosis, *N*-acetylcysteine, Minocycline, Neuropsychiatric disorders

## Abstract

In the first part, the following mechanisms involved in different forms of cell death are considered, with a view to identifying potential therapeutic targets: tumour necrosis factor receptors (TNFRs) and their engagement by tumour necrosis factor-alpha (TNF-α); poly [ADP-ribose] polymerase (PARP)-1 cleavage; the apoptosis signalling kinase (ASK)-c-Jun N-terminal kinase (JNK) axis; lysosomal permeability; activation of programmed necrotic cell death; oxidative stress, caspase-3 inhibition and parthanatos; activation of inflammasomes by reactive oxygen species and the development of pyroptosis; oxidative stress, calcium dyshomeostasis and iron in the development of lysosomal-mediated necrosis and lysosomal membrane permeability; and oxidative stress, lipid peroxidation, iron dyshomeostasis and ferroptosis. In the second part, there is a consideration of the role of lethal and sub-lethal activation of these pathways in the pathogenesis and pathophysiology of neurodegenerative and neuroprogressive disorders, with particular reference to the TNF-α-TNFR signalling axis; dysregulation of ASK-1-JNK signalling; prolonged or chronic PARP-1 activation; the role of pyroptosis and chronic inflammasome activation; and the roles of lysosomal permeabilisation, necroptosis and ferroptosis. Finally, it is suggested that, in addition to targeting oxidative stress and inflammatory processes generally, neuropsychiatric disorders may respond to therapeutic targeting of TNF-α, PARP-1, the Nod-like receptor NLRP3 inflammasome and the necrosomal molecular switch receptor-interacting protein kinase-3, since their widespread activation can drive and/or exacerbate peripheral inflammation and neuroinflammation even in the absence of cell death. To this end, the use is proposed of a combination of the tetracycline derivative minocycline and *N*-acetylcysteine as adjunctive treatment for a range of neuropsychiatric disorders.

## Introduction

Apoptosis and necrosis are two forms of cell death. Apoptosis relies on an intracellular proteolytic cascade which is essentially mediated by two types of caspases (named after the fact that they are proteases with cysteine at the active site and with aspartate targets), namely initiator caspases (such as caspase-8 and caspase-9) and executioner caspases (caspase-3, caspase-6 and caspase-7). Two important mammalian pathways which can activate an initiator caspase are the extrinsic pathway and the intrinsic (or mitochondrial) pathway [1]. Extrinsic apoptosis is activated by the binding to death receptors of members of the tumour necrosis factor (TNF) superfamily of cytokines, such as TNF-α, fibroblast-associated cell surface (Fas) ligand (FasL) and TNF-related apoptosis-inducing ligand (TRAIL). In turn, this induces the formation of the death-induced signalling complex (DISC), which in turn activates caspase-8 (an initiator caspase). Activated caspase-8 activates caspase-3 (an executioner caspase), which executes apoptosis. The activation of the extrinsic apoptotic pathway can lead to the activation of the intrinsic apoptotic pathway, which is dependent on the activity of mitochondria [2], whereby activated caspase-8 cleaves the pro-apoptotic Bcl2 homology (BH) domain BH-3-only protein Bid that induces outer mitochondrial membrane permeabilisation through the interactions of truncated Bid (tBid) with the pro-apoptotic effector Bcl2 family proteins Bax/Bak, resulting in the mitochondrial release of apoptogenic cytochrome *c*, and other apoptosis inducing factors, such as the second mitochondria-derived activator of caspases/direct inhibitor of apoptosis (IAP)-binding protein (SMAC/DIABLO) [3] into the cytoplasm. Translocation of cytochrome *c* into the cytosol in turn initiates the assembly of the heptameric apoptosome, containing Apaf-1 and procaspase-9, resulting in the cleavage of the latter and subsequent downstream activation of caspase-3 and apoptotic death [4]. Notably, the intrinsic pathway of apoptosis can be activated following oxidative damage to mitochondrial proteins, DNA damage and peroxidative damage to cardiolipin and other mitochondrial membrane lipids driven by excessive levels of reactive oxygen species (ROS) and reactive nitrogen species (RNS). It is also important to note that TNF-α engagement with TNF receptors (TNFRs) can be anti-apoptotic in certain circumstances and that several mechanisms driving caspase-independent apoptosis also exist, which will be discussed below [5].

Given the acknowledged role of elevated TNF-α, ROS and RNS in the pathogenesis and pathophysiology of neurodegenerative disorders, it is unsurprising that copious evidence exists describing activated or disorganised apoptotic pathways and increased caspase activity in these conditions [6–8]. There is also evidence of activated apoptotic programmed cell death pathways in the frontal cortex of patients with major depressive disorder (MDD), and in the anterior cingulate cortex (ACC) and hippocampus of patients with bipolar disorder (BD) and schizophrenia, which are increasingly described as neuroprogressive conditions [9–11]. This form of cell death is held to be responsible for the reduced neural and glial density seen in the dorsolateral prefrontal cortex of MDD patients [12] and in the ACC and hippocampus of patients with BD and schizophrenia [13, 14].

Several research teams have demonstrated the existence of ferroptosis, parthanatos, pyroptosis, necroptosis and lysosomal membrane permeability (LMP) as drivers of necrotic cell death in at least some cells and tissues in Parkinson’s disease (PD), Alzheimer’s disease (AD) and other neurodegenerative diseases [15–19]. There is also some evidence implicating lysosomal rupture in the formation of amyloid plaques, especially in AD [20]. This is unsurprising given that high levels of TNF-α, oxidative and nitrosative stress characteristic of neuroprogressive and neurodegenerative conditions (reviewed in references [6, 7, 21]) are known to activate poly [ADP-ribose] polymerase (PARP)-1 and the Nod-like receptor NLRP3 inflammasome, provoke LMP, precipitate necroptosis and exacerbate the development of ferroptosis [22–24].

There are numerous studies demonstrating that widespread sub-lethal activation of the TNF-α, PARP-1 and NLRP3 signalling pathways, the presence of lysosomal dysfunction, iron accumulation, lipid peroxidation and downregulation of positive inhibitors of ferroptosis singly and collectively play a causative role in the pathophysiology and pathogenesis of many if not all neurodegenerative diseases [19, 25, 26]. Activation of PARP-1 signalling, the NLRP3 inflammasome, TNF-mediated inflammatory pathways, lysosomal dysfunction, lipid peroxidation and downregulation of positive inhibitors of ferroptosis have also been repeatedly demonstrated in neuroprogressive disorders [10, 27–32]. There is also evidence of iron dyshomeostasis in MDD and BD [33, 34], and researchers have previously observed cellular iron accumulation in at least some patients [35, 36]. The situation in schizophrenia, however, is less clear. This is predominantly because of the confounding effects neuroleptics have on the mechanisms of iron homeostasis [37]. Iron accumulation in neurones and microglia of patients with neuroprogressive conditions would not be unexpected however given that elevated levels of oxidative stress and neuroinflammation characteristic of these conditions would be expected to provoke iron dyshomeostasis. Such dysregulation could lead to iron accumulation in microglia and neurones in at least some regions of the brain [38–40].

Widespread activation of TNF-α, PARP-1, NLRP3 and the necrosomal molecular switch receptor-interacting protein kinase-3 (RIPK3) in patients with neurodegenerative and neuroprogressive diseases may be of importance from the perspective of pathogenesis and pathophysiology as increased activity of these molecular players can drive and/or exacerbate peripheral inflammation and neuroinflammation even in the absence of cell death [25, 41–43]. This may be especially important in neuroprogressive disorders given chronic neuroinflammation—characterised by activated microglia—is an acknowledged source of impaired adult neurogenesis and abnormalities in neurotransmitter systems, which are a recognised source of pathology [29, 44, 45]. Targeting the amelioration of these signalling pathways would seem to be a desirable therapeutic objective in addition to attempting to reduce oxidative stress and inflammation. Accordingly, this review has three objectives: First, to explain the mechanisms involved in the various forms of cell death with a view of identifying therapeutic targets; second, to outline the potential role that sub-lethal and lethal activation of these pathways could play in the pathogenesis and pathophysiology of neurodegenerative and neuroprogressive illnesses; finally, to propose a combination of minocycline and *N*-acetylcysteine (NAC) as an adjunctive treatment for neuroprogressive conditions focusing on their role in inhibiting molecular players involved in a multitude of cell death pathways.

Apoptosis and necrosis are viewed as opposite extremes on a spectrum of cell death and can occur simultaneously in the same tissue [46, 47]. Necrosis can also take place as a “backup” form of cell death upon failure of caspase-dependent apoptotic mechanisms [48, 49]. This is of importance as levels of TNF-α and ROS play a significant role in determining whether programmed cell death proceeds via apoptosis, whereby excessive levels tend to promote cell death via necrosis while inhibiting apoptosis [46, 47]. A major mechanism driving this preference for necrotic cell death in an environment of prolonged and/or excessive inflammation and oxidative stress is the inhibition of caspase activity and the subsequent collapse of cellular adenosine triphosphate (ATP) generation [50]. This inhibition occurs because the activity of caspases is dependent on the presence of thiol groups in their catalytic sites which are an indispensable element in their capacity to act as proteases and the fact that these groups are exquisitely sensitive to oxidative or nitrosative inactivation in an environment characterised by very high levels of ROS and NO (reviewed by reference [1]). Mechanistically, this phenomenon is underpinned by the inhibition of processes such as proteasome-mediated degradation and gene translation, which consume large amounts of ATP by caspase activation following the instigation of apoptotic machinery [50, 51]. With the inhibition of caspase activity, these processes continue unchecked leading to the collapse of cellular ATP levels which prompts the switch from cell death by apoptosis to cell death by necrosis [50]. One well-documented example of this phenomenon is the inhibition of caspase-8 activity in complex II formed during TNF-α-mediated apoptosis which switches the mode of cell death to TNF-α-mediated necroptosis, while another is the inhibition of caspase-3 which leads to hyperactivation of PARP-1 inducing the development of parthanatos (also known as PARP-1-dependent cell death) [49]. We will now move on to consider these processes in some more detail before discussing other forms of cellular necrosis such as pyroptosis, lysosome-mediated necrosis and ferroptosis.

## Mechanisms Underpinning TNF-α- and ROS-Mediated Apoptosis

### Engagement of TNFRs by TNF-α

Following stimulation with TNF-α, the TNF receptor TNFR1 translocates into lipid rafts and recruits TNFR-associated protein 2 (TRAF2), the adapter protein TNFR1-associated death domain protein (TRADD), cellular inhibitor of apoptosis protein (cIAP), the E3 ligase linear ubiquitin chain assembly complex (LUBAC) and, finally, receptor-interacting protein kinase-1 (RIPK1) to the plasma membrane, resulting in the formation of a transient signalling platform generally described as complex I [52]. Once in situ, RIPK1 is multiply ubiquitinated and phosphorylated by the E3 ligase LUBAC and cIAP within lipid rafts, leading to rapid activation of nuclear factor-κB (NF-κB) [53, 54] and several downstream anti-apoptotic proteins, such as cellular FLICE (Fas-associated death domain-like IL-1β-converting enzyme)-like inhibitory protein (cFLIP) [52]. Importantly, the ubiquitination status of RIPK1 is an essential element in maintaining NF-κB activation and the stable anchorage of the kinase at the plasma membrane [52, 55]. As such, RIPK1 deubiquitination is mediated by a range of multiple deubiquitinating enzymes (DUBs) such as A20 and cylindromatosis (CYLD), which are upregulated by NF-κB at excessive intracellular concentrations of TNF-α [56, 57]. This inhibits NF-κB signalling, leading to the dissociation of the kinase into the cytoplasm to act as the initial recruiting molecule for the formation of a cytosolic DISC generally described as complex II. This cytosolic signalling platform is comprised of RIPK1, caspase-8, FADD and cFLIP recruited from the cytosol [52, 58]. Here, levels of caspase-8 are deterministic of downstream events. Adequate levels of caspase-8 activate caspase-3 and further downregulate NF-κB signalling by proteolytic cleavage of RIPK1 [59].

### Role of PARP-1 Cleavage

Caspase-8 and FADD in tandem inhibit necroptosis by inhibiting the activity of RIPK1 and RIPK3 and CYLD and promote the advent of apoptosis via the cleavage of PARP-1 [58, 60]. Cleavage of PARP-1 by caspase-3 is held to be a universal hallmark of apoptotic cell death [61, 62]. Interestingly, virtually every caspase displays the capacity to inhibit PARP-1, albeit in vitro [63]. Cleavage of PARP-1 by caspase-3 leads to the formation of two catalytic subunits of 89 and 24 kDa with the former exiting into the cytosol and the latter being retained in the nucleus [64, 65] where its binding to DNA inhibits the activity of PARP-1 abrogating DNA repair but conserving levels of ATP, thereby favouring the development of apoptosis [66].

### Role of the ASK-JNK Axis

TNF-α-mediated apoptosis is also dependent on ROS production following TNFR engagement [67] and subsequent activation of apoptosis signalling kinase-1 (ASK-1). Briefly, elevated levels of ROS provoke the disengagement of thioredoxin which is attached to ASK-1 in physiological conditions and acts to inhibit its activity [68]. Once activated, this kinase phosphorylates the downstream mitogen-activated protein kinase (MAPK) c-Jun N-terminal kinase-1 (JNK1), which is the effector molecule of an apoptotic cascade [69–71]. The sustained apoptotic activity of JNK1 is dependent on TRAF2 and RIPK1 activity and the formation of a TRAF2/RIPK1/JNK1 signalling complex at the plasma membrane [72]. Mechanistically, RIPK1 and TRAF2 are recruited to lipid rafts in an environment of chronic oxidative stress where they associate with JNK1 recruited from the cytoplasm [72–74]. Once endocytosed, this signalling complex enables the detrimental effects of JNK on mitochondrial membranes to take place and increases the activity of PARP-1, leading to nicotinamide adenine dinucleotide (NAD^+^) depletion in the cytosol [74, 75].

### Role of Increased Lysosomal Permeability

TNF-α-mediated apoptotic signalling triggers lysosomal permeabilisation and the subsequent release of lysosomal proteinases known as cathepsins into the cytoplasm; this involvement of cysteine-dependent cathepsins in the cytoplasm secondary to the development of lysosomal permeabilisation plays a significant role in the development of apoptosis [76–78]. Some debate remains as to the major mechanisms underpinning the pro-apoptotic role of cathepsins, but the weight of evidence suggests that their role in caspase-dependent apoptosis may be limited to caspase activation while cathepsins B, L and D would appear to be pivotal players in instigating or exacerbating caspase-independent cell death in the brain [79]. Some cathepsins, such as cathepsin D, have the capacity to induce mitochondrial membrane permeabilisation and mitochondrial permeability transition via proteolytic activation of the Bcl-2 family members Bid, Bax and Bak. This results in Bax/Bak activation, the formation of pores in the outer mitochondrial membrane and the escape of cytochrome *c*, apoptosis-inducing factor (AIF) and SMAC/DIABLO from the inter-membrane space into the cytoplasm triggering apoptotic death [80–82].

## Mechanisms Underpinning Non-apoptotic Programmed Cell Death

### Increased Levels of ROS and TNF-α and the Activation of Programmed Necrotic Cell Death

In an environment where caspase-8 activity (in complex II, formed following TNF ligation of TNFR; as described above) is inhibited by cIAP, increased oxidative stress or post-translational modifications [1], complex II is unable to initiate intrinsic apoptosis or inhibit the activity of RIPK1 and RIPK3 [83]. In such circumstances, RIPK1 and RIPK3 autophosphorylate and transphosphorylate with one another to form a complex described as the necrosome. In addition to containing RIPK1 and RIPK3, this cytosolic structure also includes cIAP and the mixed lineage kinase domain-like protein (MLKL) [84, 85]. The conditional DUBs CYLD and A20 deubiquitinate RIPK1 [52, 86, 87]. RIPK1 subsequently phosphorylates RIPK3, which in turn phosphorylates MLKL leading to its oligomerisation [87, 88]. This change in MLKL conformation triggers recruitment to the plasma membrane. Once in situ, it obtains anchorage by binding negatively charged phosphatidylinositol phosphate (PIP), before translocating into lipid rafts. Here, it induces the type of necrosis known as necroptosis, by triggering Na^+^ and Ca^2+^ influx into the cell [87].

Considerable evidence now exists suggesting that ROS have a direct role in driving necroptosis and apoptosis via routes which are independent of caspase activation [89]. For example, several research teams have reported that excessive levels of TNF can induce a large increase in mitochondrial ROS production which enhances necrosome formation (see, for example, reference [90]). Indeed, recent evidence suggests that physiologically elevated levels of mitochondrial ROS production are an essential element in RIPK3 recruitment into the necrosome [91]. The indispensable role ROS play in enabling or driving TNF-α-induced apoptosis or necroptosis is further emphasised by data demonstrating that these forms of cell death are inhibited by the administration of free radical scavengers even in an environment where extracellular and intracellular TNF-α levels are excessive [92, 93] (review by reference [91]).

### Increased Oxidative Stress, Caspase-3 Inhibition and Development of Parthanatos

Increased levels of oxidative stress and the subsequent inhibition of caspase-3 lead to persistent PARP-1 hyperactivation. This promotes excessive consumption of NAD^+^ and the exhaustion of cellular ATP, leading to necrotic cell death [94–96]. Mechanistically, this form of caspase-independent programmed necrosis involves the sequential activation of PARP-1, calpains, Bid- and Bax-induced mitochondrial membrane depolarisation, mitochondrial permeability transition pore opening and efflux of AIF into the cytoplasm and ultimately the nucleus [97, 98]. Once in the nucleus, AIF associates with the phosphorylated histone H2AX to form a DNA-degrading complex that induces chromatinolysis and cell death by parthanatos [99]. Importantly, while direct depletion of NAD^+^ by PARP-1 is responsible for a catastrophic decline in ATP generation and efflux of AIF is mediated by calpain, the initial signalling of PARP-1 to mitochondria is mediated by the downstream activation of JNK1 [100] (review by reference [101]).

### Activation of Inflammasomes by ROS and the Development of Pyroptosis

ROS can activate the complex cytosolic multiprotein signalling platforms known as inflammasomes [23, 102]. Typically, these structures are composed of sensor and adaptor proteins and the zymogen procaspase-1, with the latter processed into active caspase-1 following inflammasome complex assembly [103]. Caspase-1 in turn cleaves the zymogen forms of interleukins IL-1β and IL-18 leading to their activation and the development or exacerbation of inflammation and in many cases pyroptotic cell death [103, 104]. Inflammasome activation can also lead to pyroptotic cell death via caspase-1- and caspase-11-mediated cleavage of the cytoplasmic protein gasdermin D (Gsdmd) and other cell death mediators whose actions are not currently understood [105, 106]. Pyroptosis induces rapid plasma membrane rupture and excessive release of pro-inflammatory cytokines (PICs) and chemokines which may aggravate inflammation-mediated neuronal death [107, 108]. These molecular players are also mediators of activated leucocyte recruitment from the periphery which exacerbate inflammatory responses causing severe central nervous system (CNS) tissue damage in neuropathological conditions. As these factors have been shown to mediate the recruitment of other immune cells from the peripheral circulation, an abundance of leukocytes is attracted to the inflammation sites, and subsequent inflammatory responses can cause severe tissue damage in the CNS under neuropathological conditions [109].

In the case of activation of the inflammasomes NLRP3 and absent in melamoma-2 (AIM-2) however, the execution of this form of lytic and inflammatory cell death is not inevitable, as mechanisms exist that can inhibit the signalling of and accelerate the clearance of these complexes [110]. Notably, chronic low level activation of NLRP3 plays a major causative role in the pathogenesis and pathophysiology of a range of autoimmune, metabolic, neurological and neuroprogressive illnesses (review by references [111, 112]). The assembly and performance of inflammasomes is regulated by an almost bewildering array of enzymes, including, but not limited to inhibitory kappa-B kinase-1 (IKK1), IKK2, LUBAK and JAK1 (see reference [110]). Importantly, given the levels of nitrosative and oxidative stress seen in patients with neuroprogressive disorders, inflammasome activity can also be regulated by levels of nitric oxide-induced protein S-nitrosylation [113]. This is also true of caspase-1 activity, which may be inactivated in such a cellular environment [114] and which may also partly explain how NLRP3 activation in these circumstances may have a range of sub-lethal consequences in at least some regions and tissues in the brain. While oxidative stress clearly plays a role in inflammasome activation, high levels of ROS and NOS and/or the development of calcium dyshomeostasis can also induce LMP in some circumstances, leading to another “explosive” form of cell death described as lysosomal-mediated necrosis (LMN), described in the following section.

### Oxidative Stress, Calcium Dyshomeostasis and Iron in the Development of LMN and LMP

LMN is another form of cell death largely mediated by the lethal proteolytic effects of cysteine cathepsins entering the cytosol following complete lysosomal membrane rupture [115–117]. This form of cell death is characterised by the proteolysis of several crucial inflammatory protein zymogens, such as caspase-1 and IL-1, and severe damage to the cell plasma membrane via indeterminate mechanisms [117]. Lysosomal rupture may also occur as an upstream event in at least some forms of apoptosis [118, 119]. The weight of evidence would suggest that the degree and perhaps the speed of lysosomal rupture may be decisive with moderate lysosomal rupture inducing apoptosis, while pronounced lysosomal leakage results in necrosis devoid of caspase activation [120]. The mechanisms underpinning this dichotomy are not fully understood, but accumulating evidence suggests that the massive influx of low molecular mass iron into the cytosol secondary to complete lysosomal rupture results in the inactivation of functional cysteine thiol groups in the catalytic site of procaspase-9, thereby preventing its activation [121, 122].

Several members of the cathepsin family are involved in mediating LMN depending on the nature of cytotoxic stimuli involved. For example, cathepsins S and B enable alum (aluminium hydroxide)-mediated necrosis and the development of adaptive immunity following immunisation [123–125], while cathepsin D mediates LMN following lysosomal permeability driven by high levels of intralysosomal iron [126]. Given the redox-active nature of intralysosomal iron ions, and their ability to engage in the Fenton reaction with hydrogen peroxide, it is unsurprising that rapid release of intralysosomal iron into the cytosol following lysosomal rupture is a significant driver of cell death, either alone or in synergy with other oxidative drivers of DNA damage and LMP [127–129]. Increased iron accumulation within cellular lysosomes and in other cellular compartments is held to be a major contributing factor in ferroptosis. In this context, it is noteworthy that high levels of ROS, RNS and TNF-α are key drivers of iron dyshomeostasis, which consequentially leads to intracellular iron accumulation [39, 130, 131]. There are many factors involved in the development of ferroptosis outside of iron dyshomeostasis however, and this form of cell death appears to be unique in that genetic susceptibility seems to have a major influence. We will consider this element, as well as the other factors driving ferroptotic cell death in the next section.

### Oxidative Stress, Lipid Peroxidation, Iron Dyshomeostasis and Ferroptosis

Ferroptosis is characterised by the accumulation of iron and lipid hydroperoxides and their metabolites in the cytosol and is effected by fatal peroxidation of polyunsaturated fatty acids (PUFAs) in the plasma membrane [132–135]. There is considerable evidence implicating disturbed iron homeostasis in ferroptosis; for example, impaired iron regulatory protein 2 (IRP2) activity coupled with unusually high levels of the transferrin receptor, transferrin and mitochondrial ferritin has been linked with this process [88, 132, 136]. Some other elements which appear to be peculiar to the process of ferroptosis include the need for active lysosomes [137] as well as the involvement of mitochondrial glutaminase-2 and subsequent glutaminolysis [26, 132, 138]. In addition, several mitochondrial genes are associated with the development of this form of cell death, and there is evidence suggesting peroxidation of cardiolipin [26, 139]. Despite this, mitochondrial ROS, calcium dyshomeostasis and the interaction of truncated Bid, Bax and Bad do not appear to be triggers of ferroptosis [24, 140].

Recent evidence also indicates that the increased activity of certain types of lipoxygenase (LOX), most notably 15-LOX, and subsequent oxidation of arachidonic acid and phosphatidylethanol are indispensable elements in the induction of ferroptotic cell death [134, 141, 142]. Ferroptosis is negatively regulated by nuclear factor (erythroid-derived 2)-like 2 (Nrf2 or NFE2L2 or NF-E2-related factor 2), glutathione, glutathione peroxidase-4 and the glutamate/cystine antiporter system and positively regulated by NADPH oxygenase and p53 [26]. Lipoxygenase-mediated peroxidation of PUFAs, with the resultant production of oxidised phosphatidylethanolamine and the two fatty acyls, arachidonoyl and adrenoyl, coupled with the mediation by the acyl-CoA synthetase long-chain family member ACSL4 of the production of 5-hydroxyeicosatetraenoic acid, would appear to be the ultimate executioners of ferroptosis [134, 142, 143]. It would also appear that the lipid composition of the lipid membrane is an important factor, with an increased concentration of long-chain omega-6 PUFAs conveying particular risk [144]. This is important given evidence now suggests that the level of ACSL4 might well dictate the sensitivity towards the development of ferroptosis by influencing the lipid composition of the cell membrane [144]. Individual differences in the activity of ACSL4 might in part explain the strong genetic component in the sensitivity to ferroptosis, and manipulation of this enzyme may ultimately offer an appropriate therapeutic intervention [141, 144].

The role of iron in this process remains a matter of debate [145]; however, iron dyshomeostasis would appear to be involved, and there is evidence that ferroptosis includes ferritinophagy and subsequent release of redox-active iron into the cytosol via the nuclear receptor co-activator 4 (NCOA4)-regulated autophagy pathway [26, 143]. There is also some evidence that iron-mediated activation of phosphorylase kinase G2 (PHKG2) directly mediates lipoxygenase-mediated peroxidation of PUFAs leading to the accumulation of lethal hydroperoxides, but this datum is yet to be replicated [134]. Finally, a recent study conducted by Muller and colleagues produced data suggesting that ferroptosis and necroptosis are two alternative cell death pathways and inhibition of one provokes induction of the other [146]. The mechanisms underpinning this phenomenon are yet to be delineated however.

Having examined the mechanisms involved in cell death mediated by TNF-α, ROS and RNS, we now turn our attention to the second objective of the paper, namely an examination of the potential roles of these cell death pathways both at lethal and sub-lethal levels of activation in the pathogenesis and/or physiology of neuroprogressive and neurodegenerative conditions.

## Putative Involvement of Cell Death Machinery in the Pathogenesis and Pathophysiology of Neurodegenerative and Neuroprogressive Illnesses

### The TNF-α-TNFR Signalling Axis

High levels of TNF-α are found in the brains and cerebrospinal fluid (CSF) of patients with PD and AD and are implicated in the pathogenesis and pathophysiology of both illnesses [147–150]. Consistent with this, increases in TNFR1 activity and decreases in TNFR2 activity have also been frequently reported in both diseases [150–152]. There would appear to be a difference in the distribution of this abnormal brain TNFR activity in these two conditions however, with this phenomenon being widespread in AD patients but confined to the substantia nigra in patients diagnosed with PD [152]. The importance of increased TNF-α and TNFR1 activity in the pathogenesis of AD is highlighted by data suggesting that levels of TNFR1 activity are predictive of the transition between mild cognitive impairment and AD [153]. It is also interesting to note that TNFR1 activity is predictive of the development of neurocognitive disturbance in PD, but there would appear to be no published research investigating TNFR1 activity and the severity of motor symptoms [154]. There would also appear to be a dearth of data investigating TNFR activity in other chronic neurodegenerative diseases, although levels of TNFR1 and TNFR2 activity are abnormal in the animal model of multiple sclerosis (MS), described as experimental autoimmune encephalomyelitis [152].

Elevated TNF-α levels and TNFR1 activity are also seen in the brain and periphery in MDD, and the connection between TNF signalling and illness progression and severity is well documented [155, 156]. Interestingly, while TNF-α levels are elevated in all phases of BD, TNFR activity would appear to differ from that observed in MDD with elevations in the levels of TNFR1 and TNFR2 activity being a replicated finding even in patients during the euthymic phase of the illness [157]. Unsurprisingly, TNFR1 activity is also elevated in patients who have received a diagnosis of schizophrenia [158], and there are data demonstrating that increased levels of TNF-α are associated with acute exacerbations of this illness [159].

### Dysregulation of ASK-1-JNK Signalling

Dysregulated JNK signalling is implicated in the pathophysiology of PD [160] and AD [161], by facilitating dopaminergic neuronal death and modulating the activity of p53 upregulated modulator of apoptosis (PUMA), respectively [162]. As well as regulating neuronal apoptosis, JNKs also regulate brain morphogenesis and the architecture of dendrites during neural development and govern crucial neurone-specific activities such as the formation of long-term memory and synaptic plasticity (review by reference [163]). Data implicating JNK1 in the regulation of dendrite arborisation in the cerebellum [164] and hippocampus [165] are of particular interest as overgrowth of dendrites during development is associated with the pathogenesis of schizophrenia and autism spectrum disorders [166]. Moreover, there is direct evidence of JNK1 signalling abnormalities in both conditions [167, 168]. For example, genetic risk for schizophrenia is associated with the JNK pathway [168], and the activity of JNK1 in the cerebral cortex is heavily reliant on a kinase with genetic locus 16p11.2, a well-documented genetic susceptibility locus for schizophrenia and indeed autism [167, 169]. Furthermore, the *interleukin-1 receptor accessory protein like-1* gene, implicated in autism, transduces signals via JNK activation [170]. It is also worthy of note that loss of function of some JNK family members owing to chromosomal translocations is associated with the development of intellectual disability [166, 171].

ASK-1 is activated by oxidative stress, nitrosative stress, endoplasmic reticulum (ER) stress, PICs, lipopolysaccharide (LPS) and Ca^2+^ influx [172] and plays a pivotal role in cell differentiation and the development of chronic inflammation as well as having a well-documented role in driving apoptosis [173]. This kinase acts as a major vehicle for the direct transduction of ROS signalling to downstream targets, and its level of activity influences both the rate of progression and the severity of several neurodegenerative diseases [174, 175]. For example, an amyloid beta-mediated increase in ASK-1-JNK1 activity appears to be an important element driving neuronal death in AD [176], while ASK-1-JNK1-mediated dopaminergic neuronal death appears to be involved in the pathogenesis of PD [177]. More generally, ASK-1 activation plays a significant role in driving the pathophysiology of numerous diseases involving grossly dysfunctional cellular responses to the advent of ER stress and oxidative stress [178].

### Prolonged or Chronic PARP-1 Activation

Unsurprisingly, several research teams have adduced evidence of caspase-3-mediated cleavage of PARP-1 in a number of neurological conditions such as MS and AD, and the process is also implicated in the development of *N*-methyl-d-aspartate (NMDA) receptor-mediated excitotoxicity (review by reference [179]). PARP-1 mediates the transfer of poly-adenosine diphosphate (poly-ADP) from NAD^+^ to DNA thereby provoking chromatin remodelling and changes in DNA methylation and histone acetylation and thus acting as an epigenetic regulator of gene transcription. Furthermore, PARP-1 also mediates the transient attachment of poly-ADP to target proteins thereby acting as a post-translational regulator of protein function [180, 181]. From the perspective of this paper, it is of particular interest that PARP-1 activity regulates the activation levels and differentiation patterns of T and B lymphocytes, via the regulation of transcription factors such as nuclear factor of activated T cells (NFAT) and the activity of inflammatory pathways in response to acute or chronic cellular stressors [182]. In particular, increased PARP-1 activity sustains the production and activity of PICs such as TNF-α and IL-1β, chemokines such as macrophage inflammatory protein (MIP)-2 (also known as chemokine (C-X-C motif) ligand 2 (CXCL2)), and selectins in the periphery and in glial cells of the brain [183].

Apart from its well-documented role in DNA damage repair, PARP-1 modulates many processes in glial cells contributing to the development of neuroinflammation [184, 185]. For example, PARP-1 is an indispensable binding partner in the NF-κB-mediated activation of microglia and in enabling the transcription of inflammatory molecules such as IL-1β, TNF-α and NO [184, 186]. PARP-1 also regulates the activation of astrocytes and their production of inflammatory cytokines and chemokines [185]. Furthermore, the activation of PARP-1 in astrocytes leads to profound bioenergetic depletion in these glia and subsequent inhibition of glutamate reuptake, thereby contributing to the development of NMDA receptor excitotoxicity, which is a feature of neuroprogressive diseases [187]. PARP-1 also exerts a number of physiological roles in the CNS and its levels are regulated by neural activity [188, 189]. The weight of evidence suggests that this transcription factor plays an important role in the consolidation and reconsolidation of long-term memory and is particularly involved in the extinction of contextual fear memory [190].

### Role of Pyroptosis and Chronic Inflammasome Activation

Pyroptosis has been observed in microglia, astrocytes and neurones [191, 192]. Moreover, several authors have reported the involvement of abnormal levels of NLRP3 signalling in a wide range of neurological disorders [193–195]. For example, increased activity of NLRP3 and caspase-1 in the brains of AD patients has been repeatedly reported [194, 196]. Similarly, other research teams have reported increases in the expression and activity of NLRP3, IL-1β, IL-18 and caspase-1 in plaques of MS patients and the tissues of amyotrophic lateral sclerosis (ALS) (or motor neurone disease) patients [195, 197–199].

Increased levels and activity of caspase-1, IL-1β and IL-18 have also been recorded in several disorders characterised by the existence of chronic neuroinflammation [200–202]. Importantly, from the perspective of generating pathology, both IL-1β and IL-18 bind to their respective cognate receptors on neurones, microglia and astrocytes, thereby triggering a highly complex pattern of inflammatory signalling pathways culminating in increased transcription of pro-inflammatory genes [19]. The activation of these pathways and increased transcription of these genes are also associated with the development of cognitive decline and the development of long-term neuroprogressive illnesses [203]. Additionally, there is evidence suggesting that NLRP3 activation in microglia is a driver of neuroinflammation in MDD [28, 204] and a source of chronic immune activation and mitochondrial complex I dysfunction seen in the brains of patients with BD and schizophrenia [205, 206]. Finally, it should be emphasised that NLRP3 activation is increasingly recognised as being a causative factor in the inflammation, mitochondrial dysfunction and chronic oxidative stress seen in autoimmune and autoinflammatory diseases (review by reference [207]).

### Role of Lysosomal Permeabilisation

LMN is another form of cell death largely mediated by the lethal proteolytic effects of cysteine cathepsins entering the cytosol following the development of lysosomal permeabilisation [115–117]. LMN is characterised by proteolysis of several crucial (pro-)inflammatory zymogens such as caspase-1 and IL-1 and severe damage to the cell plasma membrane via unclear mechanisms [117]. Several members of the cathepsin family are involved in mediating LMP depending on the nature of the cytotoxic stimuli involved. For example, cathepsins S and B enable alum-mediated necrosis and the development of adaptive immunity following immunisation [123–125]. In the absence of such external stimuli, however, lysosomal permeabilisation is mediated by high levels of oxidative stress, increased activity of calpains and unusually high lysosomal iron content [126, 208, 209]. Recent evidence also indicates that LMP in vivo is dependent on the activity of the protein signal transducers and activators of transcription-3 (Stat3) [210]. The importance of intralysosomal iron load in this form of cell death is emphasised by data revealing that cathepsin D mediates cell death following LMP driven by high levels of intralysosomal iron [126]. This is consistent with the weight of evidence indicating that that rapid release of intralysosomal iron into the cytosol following such lysosomal rupture is a major driver of cell death either alone or in synergy with other oxidative drivers of DNA damage and lipid membrane permeabilisation [128, 211, 212].

LMP is a potentially lethal event because the ectopic presence of lysosomal proteases in the cytosol causes the digestion of vital proteins and the activation of additional hydrolases including caspases. The latter process is usually mediated indirectly, through a cascade in which LMP causes the proteolytic activation of Bid (which is cleaved by the two lysosomal cathepsins B and D). Bid activation then induces mitochondrial outer membrane permeabilisation, resulting in cytochrome *c* release and apoptosome-dependent caspase activation. However, massive LMP often results in cell death without caspase activation; this cell death may adopt a subapoptotic or necrotic appearance [209].

Perhaps the strongest evidence of the involvement of frank lysosomal rupture in the pathogenesis of a neurodegenerative disease has been provided by the finding that lysosomal dysfunction is associated with AD and that lysosomal dysfunction is also associated with both the formation of β-amyloid peptide (Aβ) and the hyperphosphorylation of tau protein; two of the most important neuropathological features of AD are amyloid plaques and neurofibrillary tangles, which are caused by dysfunction and accumulation of Aβ and abnormally phosphorylated tau, respectively (review by reference [213]). There is also some evidence to support the view that lysosomal rupture induced by α-synuclein is a cause of dopaminergic neuronal death in PD [214]. More generally, LMP is increasingly regarded as a major driver of ROS-mediated cell death [209, 215], and calpain-mediated LMP is considered to be a major element in the development of pathological lysosomal dysfunction and neuronal necrosis characteristic of most, if not all, neurodegenerative diseases [208, 216, 217] (review by reference [218]). However, there would appear to be no published data investigating the potential existence of LMP as a source of pathology in neuroprogressive disorders despite the presence of chronic oxidative stress, increased calpain activity and lysosomal dysfunction [219, 220].

### Role of Necroptosis

There is widespread evidence of necroptosis in a broad array of neurological, neuroprogressive, autoimmune and other inflammatory diseases, as this process is the source of extracellular damage-associated molecular patterns (DAMPs) such as high-mobility group box 1 (HMGB1), S100B and mitochondrial DNA seen in all these conditions [51, 221, 222]. The production of DAMPs is of prime importance as these molecules activate pathogen recognition receptors on antigen-presenting cells, leading to the chronic activation of immune and inflammatory pathways. DAMP production therefore is a major driver of the chronic inflammation, immune activation and oxidative stress seen in illnesses ranging from AD, to systemic lupus erythematosus (SLE) to MDD [223, 224]. While DAMPs play a role in the pathogenesis and pathophysiology of an extensive array of medical conditions (review by references [225, 226]), the role extracellular mitochondrial DNA plays in initiating and maintaining what has been described as the “neuroinflammation-neurodegeneration alliance”—which drives the progression of neurodegenerative diseases—is particularly pertinent for the matters considered in this paper [227, 228]. DAMPs such as mitochondrial DNA, S100B and the 70-kDa heat shock proteins (HSP70s) are also a major cause of chronic immune activation in BD [229], schizophrenia [230] and MDD [45, 223]. Defective caspase-8 activation and increased levels RIPK1, RIPK3 and MLKL have been demonstrated in the cortical lesions of MS patients post-mortem [231] and would appear to be the major driver of exocytotic neuronal death, particularly as far as hippocampal neurones are concerned [232].

### The Role of Ferroptosis

Rapidly accumulating data suggest that ferroptosis is an important mediator of cell death in PD and AD [233–235]. Furthermore, there is mounting evidence that the processes underpinning this form of cell death, such as decreased glutathione and glutathione peroxidase-4 (GPx-4) levels, are involved in the pathogenesis and pathophysiology of BD [236, 237], schizophrenia [237–239] and MDD [237, 240] (review by reference [241]). Readers interested in the classification of lipid endoperoxides and hydroperoxides and the mechanisms underpinning their production and toxic effects such as inducing lipid membrane permeabilisation, producing toxic aldehydes and acting as precursor molecules for inflammatory prostaglandins are invited to consult excellent reviews on the subject by references [242, 243].

## Minocycline and NAC as Therapeutic Inhibitors of Cell Death Machinery

### Minocycline

The capacity for minocycline to provide neuroprotection and ameliorate neuroinflammation in vivo has been established by several research teams utilising various animal models of neurodegenerative conditions, such as AD [244], PD [245], ALS [246], Huntington’s disease [247] and MS [248, 249] (review by reference [250]). Putative mechanisms underpinning these effects include the inhibition of microglial activation and proliferation [251, 252]. Unsurprisingly, there are also considerable data demonstrating that minocycline also suppresses microglial production of IL-1β, IL-6, TNF, NADPH oxidase and inducible nitric oxide synthase (iNOS), as well as inhibiting T cell egress into the brain [246, 253–255]. The mechanisms whereby minocycline therapy reduces microglial activity and neuroinflammation remain to be fully elucidated, but it would appear that inhibition of p38/MAPK and metalloproteinase-9 plays a pivotal role [251, 252, 256].

Minocycline inhibits apoptotic and necrotic cell death in vivo via a range of different mechanisms including the direct inhibition of caspase-1 and caspase-3 in the cytosol [247, 257]. Many of the anti-apoptotic effects of this tetracycline derivative occur at the level of the mitochondria. One important example is the prevention by minocycline of Ca^2+^ uptake into mitochondria, thereby preventing the development of permeability transition and the collapse of the transmembrane potential difference. This resultantly prevents the release of pro-apoptotic molecules such as cytochrome *c*, SMAC/DIABLO and AIF into the cytosol [258–260]. Minocycline also modulates levels of Bcl2 [261, 262], normalises the Bax/Bcl2 ratio [263] and also inhibits the activity of Bid, thereby preventing the downstream activation of caspases 3, 8 and 9 [8, 264–266]. It should be noted, however, that these cytoprotective effects of minocycline may be replaced by toxic effects when cells are exposed to low doses of minocycline (around 50 to 100 μM), which occur at the expense of impaired mitochondrial function and decreased ATP production at these lower concentrations, owing to minocycline-induced reductions in levels of cytochrome *c* and NAD^+^ and in activity of enzymes of the electron transport chain [267, 268].

There is also in vivo evidence suggesting that minocycline may have the capacity to mitigate pyroptosis via direct inhibition of PARP-1 activity [266, 269] as well as the ability to chelate Ca^2+^, which may suppress the activation of several, if not all, members of the calpain family [270–272]. Additionally, Shahzad and colleagues recently reported that minocycline stabilises endogenous Nrf2 by reducing its levels of ubiquitination leading to the inhibition of an NLRP3-inflammasome-induced rodent model of diabetic nephropathy. This is of interest given the acknowledged role of Nrf2 inhibition in the development of ferroptosis [24, 273]. In this context, it is also worth noting that the capacity for minocycline to inhibit the development of lipid peroxidation in vivo has also been established in several studies [274, 275]. Several research teams have also adduced evidence demonstrating that minocycline attenuates iron overload following experimental induction of intracerebral haemorrhage in rodents [138, 276, 277]. This reduction in cellular iron levels was accompanied by lowered levels of haem oxygenase-1, transferrin and non-haem iron in the brain, and ferritin in the systemic circulation. Importantly, the reduction in iron load also led to a significant reduction in objective measures of CNS damage and a significant increase in the integrity of the blood-brain barrier [138, 276, 277].

Rodent studies indicate that 50 mg/kg of minocycline has the capacity to reduce the anhedonia and sickness behaviour secondary to LPS-induced microglial activation and subsequent neuroinflammation via the reduction in levels of inflammatory mediators and in indolamine-2,3-dioxygenase (IDO) [253, 278]. This is of particular interest given the results of a recent human trial investigating the therapeutic potential of 200 mg daily minocycline for 3 months as an adjunctive treatment for MDD, which reported significant improvements in a range of clinical parameters although not in the primary endpoint [279]. These results are very similar to, although somewhat better than, those obtained by the use of NAC as an adjunctive treatment for MDD in an earlier study [280]. This may be particularly pertinent given evidence that co-administration of NAC and minocycline had synergistic effects on the attenuation of neuroinflammation in a rodent model of traumatic brain injury, superior to the either preparation alone [281]. This area appears to be worthy of further investigation and may offer a way forward where the properties unique to each molecule may provide the basis for combination therapy for MDD and possibly other neuroprogressive disorders. Accordingly, we will now discuss the possible contribution of NAC in reducing levels of ROS and TNF-α and in inhibiting various molecular players and pathways underpinning the machinery of cell death.

### NAC

Numerous researchers investigating the use of NAC supplementation in animal and human studies have published findings affirming the anti-inflammatory, antioxidant and cytoprotective properties of NAC in vivo [282–284]. These findings include increased glutathione, reduced levels of ROS as evidenced by decreased levels of hydrogen peroxide and hydroxyl radicals, reduced levels of lipid peroxidation evidenced by reduced levels of malondialdehyde and 4-hydroxy-2-*trans*-nonenal, together with restored calcium homeostasis and decreased calcium ion entry into mitochondria [285–288]. Such supplementation also leads to improved mitochondrial performance as evidenced by increased ATP production, increased mitochondrial membrane potential difference and increased outer mitochondrial membrane stability [286–288]. Further evidence of the in vivo efficacy of NAC was provided by Kose and Naziroglu [289], who reported that NAC supplementation in polycystic ovary syndrome patients was associated with reduced levels of lipid peroxidation, ROS, mitochondrial membrane depolarisation, caspase-9 and caspase-3, and increased levels of glutathione and GPx [289]. Other research teams have reported that in vivo NAC supplementation increases levels of cFLIP and cIAP and reduces levels of Bax while increasing levels of Bcl2 and decreasing translocation of cytochrome *c* and AIF into the cytoplasm, hence inhibiting many processes driving intrinsic apoptosis [286].

It would also appear that many of the biochemical consequences and symptomatic improvements produced by NAC supplementation occur via increases in the activity of the oxidative stress-inducible cystine/glutamate exchange system (system X_c_^−^) rather than merely serving as a precursor molecule providing the cysteine needed to enable increased synthesis of glutathione [290–292]. The mechanisms underpinning the NAC-induced increases in the activity of system X_c_^−^ would appear to be relatively complex and involve the stimulation of as yet undelineated cellular signalling pathways [292–294]. Predictably, the profound anti-inflammatory and antioxidant capacity of NAC has made the molecule the subject of intense research in the fields of neurology and neuropsychiatry; reviews of trials in neurology and psychiatry are given by references [295, 296].

## Conclusions

Various forms of cell death are involved in the pathogenesis and pathology of a wide range of neuropsychiatric disorders. Related signalling pathways, in addition to oxidative stress and generalised inflammatory processes, appear to offer good therapeutic targets. In particular, a combination of minocycline and NAC may offer a relatively safe and tolerable form of adjunctive treatment for such disorders, which currently can be difficult to treat.

### Authorships

All authors contributed equally to the construction and editing of the paper.
